# DNA Instability Maintains the Repeat Length of the Yeast RNA Polymerase II C-terminal Domain*

**DOI:** 10.1074/jbc.M115.696252

**Published:** 2016-03-29

**Authors:** Summer A. Morrill, Alexandra E. Exner, Michael Babokhov, Bradley I. Reinfeld, Stephen M. Fuchs

**Affiliations:** From the Department of Biology, Tufts University, Medford, Massachusetts 02155

**Keywords:** DNA structure, G-quadruplex, gene regulation, genomic instability, RNA polymerase II, transcription

## Abstract

The C-terminal domain (CTD) of RNA polymerase II in eukaryotes is comprised of tandemly repeating units of a conserved seven-amino acid sequence. The number of repeats is, however, quite variable across different organisms. Furthermore, previous studies have identified evidence of rearrangements within the CTD coding region, suggesting that DNA instability may play a role in regulating or maintaining CTD repeat number. The work described here establishes a clear connection between DNA instability and CTD repeat number in *Saccharomyces cerevisiae*. First, analysis of 36 diverse *S. cerevisiae* isolates revealed evidence of numerous past rearrangements within the DNA sequence that encodes the CTD. Interestingly, the total number of CTD repeats was relatively static (24–26 repeats in all strains), suggesting a balancing act between repeat expansion and contraction. In an effort to explore the genetic plasticity within this region, we measured the rates of repeat expansion and contraction using novel reporters and a doxycycline-regulated expression system for *RPB1*. In efforts to determine the mechanisms leading to CTD repeat variability, we identified the presence of DNA secondary structures, specifically G-quadruplex-like DNA, within the CTD coding region. Furthermore, we demonstrated that mutating *PIF1*, a G-quadruplex-specific helicase, results in increased CTD repeat length polymorphisms. We also determined that *RAD52* is necessary for CTD repeat expansion but not contraction, identifying a role for recombination in repeat expansion. Results from these DNA rearrangements may help explain the CTD copy number variation seen across eukaryotes, as well as support a model of CTD expansion and contraction to maintain CTD integrity and overall length.

## Introduction

RNA polymerase II (RNAPII)^4^ is an essential protein complex responsible for the transcription of mRNA in eukaryotes. It shares many components with other RNA polymerases, but its most distinctive feature is a C-terminal domain (CTD) in the largest and catalytic subunit Rpb1p, comprised of repeating units of a seven amino acid sequence. The CTD is essential for proper transcription and acts as a binding domain for a variety of proteins involved in transcription elongation, mRNA processing, chromatin remodeling, and DNA repair (1, 2).

In all eukaryotes, the CTD is comprised of nearly identical repeats of a seven-amino acid sequence, YSPTSPS. However, the number of CTD repeats is quite different across organisms. For example, mammals can have as many as 52 repeats. In contrast, budding yeast have only 26 repeats. Surprisingly, previous studies to understand the role of CTD repeats revealed that as few as 8 repeats will support growth (3). It is still unclear why so many CTD repeats are conserved if they are not essential for function.

Early mutational studies noted that cells could undergo spontaneous mutations within the CTD coding region to repair premature stop codons (within the repeat) that compromised function (4). Although never fully explored, these investigators observed that the DNA encoding the CTD underwent either expansion or contraction, resulting in a new sequence that produced a longer protein product. This led us to question what aspect of the CTD coding sequence was promoting DNA instability and to hypothesize that the nonessential CTD repeats primarily act as a template for DNA rearrangement.

Repetitive elements in the genome are well known to be unstable. For example, trinucleotide repeat sequences such as CAG repeats are known to form stable hairpin secondary structures that interfere with DNA replication, resulting in DNA breakage. To overcome DNA secondary structures, cells either deploy certain helicases to unwind these structures or implement DNA repair pathways to repair DNA damage associated with these noncanonical DNA conformations (5, 6). This repair process leads to changes in repeat length, which in the case of many trinucleotide repeats can lead to a number of severe neurodegenerative diseases (7).

The repetitive CTD region differs from most other repetitive regions in two important ways: 1) The coding sequence is comprised of a degenerate 21-bp repeat, and 2) it is a conserved sequence in an essential protein. It is therefore not intuitive as to what aspect of the CTD sequence might make it prone to rearrangements. Furthermore, we were interested in what potential evolutionary advantage there would be in having an unstable region within an essential protein.

Here we describe the development of a genetic system to examine DNA rearrangements in the repetitive CTD region of *RPB1*. We measure genetic expansion and contraction frequencies and identify cellular factors that regulate this process, including the recombination factor Rad52p and the G-quadruplex DNA (G_4_-DNA) associating helicase, Pif1p (8). Lastly, we have uncovered an unusual signature within the DNA of the CTD coding region that forms secondary structure *in vitro*. Based on these findings, we propose a model for CTD repeat instability and maintenance in budding yeast and speculate as to how CTD length has evolved in different organisms.

## Experimental Procedures

### 

#### 

##### Yeast Strains and Plasmids

Strains used in this study were derived from GRY3019 (*MATa his3*Δ *leu2*Δ *lys2*Δ *met15*Δ *trp1*Δ::*hisG URA*::*CMV-tTA kanRPtetO7-TATA-RPB1*) (9). The *rad52*Δ strain was constructed using heterologous gene replacement (10) and verified by PCR using primers described in Table 1. The *pif1-m2* mutation was made following the procedure described previously (11). Yeast were grown on SC dropout medium or YPD as indicated. Doxycycline (+DOX, 50 μg/ml) was added to plates to control the expression of genomic *RPB1*. When the selection drugs G418 or ClonNat were added to SC plates, ammonium sulfate was replaced by monosodium glutamate (1g/L) as a nitrogen source (12).

**TABLE 1:** **Oligonucleotides used in this work**

Sequence 5′ → 3′	Description
AGAAAAGCCTGGTGTCAAGACTCCAAACCCGGGTTGG	CTD upstream forward for RDL
AACCCGGGTTTGGAGTCTTGACACCAGGCTTTTCTCC	CTD upstream reverse for RDL
AGAATATGAAGGTGAGGTTGGGCTGTAACTAGGAGACGTCGG	CTD repeat forward for RDL
GACGTCTCCTAGTTACAGCCCAACCTCACCTTCATATTCTCC	CTD repeat reverse for RDL
AAGAGCTCATCTGGAATTTTCATTTTCATTATGCTTTTGTTCGTCTTGCTTTGGAGAATATGCAGGAGACGTCGG	CTD terminator forward for RDL
GACGTCTCCTGCATATTCTCCAAAGCAAGACGAACAAAAGCATAATGAAAATGAAAATTCCAGATGAGCTCTTCC	CTD terminator reverse for RDL
TTTACTAGCGCCGTTGGTTT	*RPB1* reverse PCR primer
GATCGATGAGGAGTCACTGG	*RPB1* forward PCR primer
ATATGCGTCAGGCGACCTCT	*RPB1* forward PCR primer
AATGCAAACAAGGAGGTTGC	*RAD52* forward primer
CGAGTACGAGATGACCACGA	NAT reverse primer
GCCAAAGTGGATAAGATCAAC	*PIF1* forward primer
TGCAATTCAGTGAAGCTAGGTC	*PIF1* reverse primer
CACCAACGTCACCATCATAATGGCCAACGTCACCATCATAT	QuikChange stop codon forward
ATATGATGGTGACGTTGGCCATTATGATGGTGACGTTGGTG	QuikChange stop codon reverse
TAATACGACTCACTATAGGG	T7 forward
TATGCTAGTTATTGCTCAG	T7 reverse
TGGTGATGTTGGGGAATAGGCTGGAGACGTTGGGT	CTD1
TTGGGGAATAGGCTGGAGACGTTGGGC	CTD2
TGGTGATGTTGGCGAGTACGATGGTGATGTTGGT	CTD3
AAAGGGTTAGGGTTAGGGTTAGGGAA	Tel26

To create repetitive CTD constructs of precise length and sequence, we used the recursive directional ligation by plasmid reconstruction (PRe-RDL) technique and plasmid JMD2 described by the Chilkoti lab (13). CTD segments were iteratively ligated together to synthesize CTD regions that were verified by sequencing. This region was amplified using T7 forward and T7 reverse primers complementary to JMD2, digested with XmaI and SacI, and subcloned into a variant of pL-RPB1 (9), which was altered by QuikChange site-directed mutagenesis to introduce unique XmaI and SacI restriction sites upstream and downstream of the CTD coding region within *RPB1*. We intended to design a CTD mutant where repeat 9 was interrupted by a stop codon that would result in a protein that could not support growth. The CTD-4stop plasmid was created by QuikChange mutagenesis using primers with homology to repeat 9 of the CTD. Because of the repetitiveness of the CTD, we were never able to isolate a mutant with a single insertion of this sequence and therefore used the isolated pRPB1–4stop plasmid for our experiments. Plasmids used in this study and relevant sequence information are listed in Table 2. CTD-containing plasmids and control vectors (14) were transformed into yeast and selected on synthetic complete medium lacking leucine (SC−Leu) (15).

**TABLE 2:** **Plasmids used in this work**

Plasmid	Reference	Notes
pL-RPB1	Ref. 9	
pRS315	Ref. 14	Referred to as pLEU in the text
pVS31	Ref. 11	Plasmid for making pif1-m2 mutation
pJMD2	Ref. 13	Plasmid for recursive directional ligation used to make CTD mutants
pSMF2	This work	pL-RPB1 with XmaI and SacI sites flanking the CTD coding region. Referred to as pRPB1 in the text
pRPB1-CTD_8_	This work	pSMF2 with 8 CTD repeats
pRPB1-CTD_10_	This work	pSMF2 with 10 CTD repeats
pRPB1-CTD_14_	This work	pSMF2 with 14 CTD repeats
pRPB1-CTD_26_	This work	pSMF2 with 26 CTD repeats
pRPB1–4stop	This work	pSMF2 with repeats 8–10 interrupted with 4 repeats containing stop codons

##### Spotting Assays

For phenotypic growth assays, yeast were grown overnight in SC−Leu to ensure retention of the plasmid copy of *RPB1* (pRPB1). Saturated cultures were used to start fresh cultures in the same medium at an *A*_600_ of 0.2. The cells were allowed to double at least two times, harvested, and resuspended to an *A*_600_ of 1.0 in sterile water in a 96-well plate. Cells were 5-fold serially diluted and then spotted onto plates containing necessary selections using a 48-pin replicating tool. Plates were incubated at indicated temperatures and imaged daily.

##### Determining Mutational Frequency of the Truncated CTD

Fluctuation analysis was modified from Aksenova *et al.* (16) to fit the DOX selection system. Briefly, strains were streaked for isolation onto SC−Leu to maintain pRPB1 and incubated at 30 °C for 3–5 days (approximately when colonies ∼10^6^ cells, which varies by strain). For each initial colony, the cells were suspended in liquid and plated for selection on +DOX medium; the same cell suspension was diluted 10^4^-fold and plated on YPD as a cell count control. For each experiment, cells derived from 12 individual colonies were plated. Suppressors were identified by colony formation on SC−Leu + DOX medium after ∼4 days. Colony counts from both the DOX selection and the matched control plates were used as input for the fluctuation analysis calculator (FALCOR), a means of calculating the mutational frequency for each strain using the MSS-maximum likelihood estimator method (MSS-MLE) (17). For mutation type analysis, a maximum of four colonies from any plate were analyzed by colony PCR. Primers flanking the CTD were used to amplify the CTD coding region using a modified PCR protocol (18). To prepare a crude DNA template from the yeast colonies, cells were lysed in 50 μl of 0.4% SDS at 90 °C for 4 min and briefly spun; 1 μl of the supernatant was used as template for a 50-μl PCR. To modify the PCR mix, we added Triton X-100 to a final concentration of 1%. Unless otherwise noted, gel electrophoresis was done using 2% agarose gels, 1× TBE, SYBR Safe DNA gel stain and run with a 100-bp ladder.

##### Western Blotting

Yeast cultures were grown in synthetic defined medium with or without DOX (50 μg/ml) from a starting *A*_600_ of between 0.1 and 0.2 and grown to mid-log phase (*A*_600_ 0.6–1.0). In all cases, five optical units of cells were harvested by centrifugation, and extracts were prepared as previously described (19). For Western analysis of Rpb1p, proteins were separated on 8% SDS-PAGE gel made with 19:1 acrylamide:bis-acrylamide. Gels were transferred to PVDF for 90 min at 45 mA and dried in methanol to block according to the manufacturer's instructions (Millipore-Immobilon-P). Dried membranes were rehydrated briefly in methanol and incubated with primary antibodies (Y-80; Santa Cruz) overnight at 4 °C in PBST (phosphate-buffered saline including 0.05% Tween 20) containing 4% milk. Western blots were visualized using HRP-conjugated secondary antibodies and ECL Plus chemiluminescence (GE Healthcare).

##### Circular Dichroism

Circular dichroism measurements were taken using a Jasco 810 instrument at 25 °C in 2 mm sodium phosphate buffer, pH 7.0, with or without 100 mm KCl. Samples were heated to 95 °C and slowly cooled to room temperature where they equilibrated for a minimum of 48 h prior to analysis. Measurements were taken at 0.5-nm intervals from 200 to 320 nm. Traces shown are the average of three measurements for each sample.

## Results

### 

#### 

##### Variability within the RNAPII CTD Sequence of Natural and Laboratory Isolates

**FIGURE 1.**
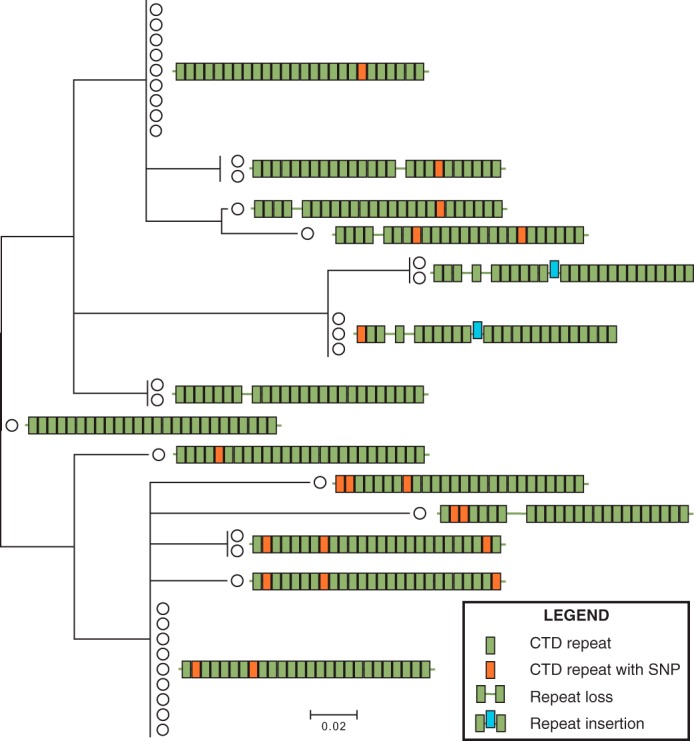
**Variability within the CTD coding sequence.** Genomic DNA from the 36 strains used in the Saccharomyces Genome Resequencing Project was sequenced by Sanger sequencing, and a multiple sequence alignment was created using MUSCLE (48). The alignment was manually adjusted to correct for repeat length changes misannotated as single nucleotide polymorphisms. Each strain is represented as a *circle* on the phylogenic tree. The schematics shows how unique 21-bp blocks (shown as *colored rectangles*) are arranged to give rise to a protein product consisting of 24–26 seven-amino acid units. *Blocks* shown in *green* represent the consensus sequence for that block position (see Fig. 2), *orange blocks* indicate the presence of a single-nucleotide polymorphism (*SNP*) within that sequence, the offset block (*blue*) indicates a 21-bp insertion unique to five *S. cerevisiae* strains, and *open gaps* in the block structure indicate that a 21-bp repeat has been lost from a particular strain.

**FIGURE 2.**
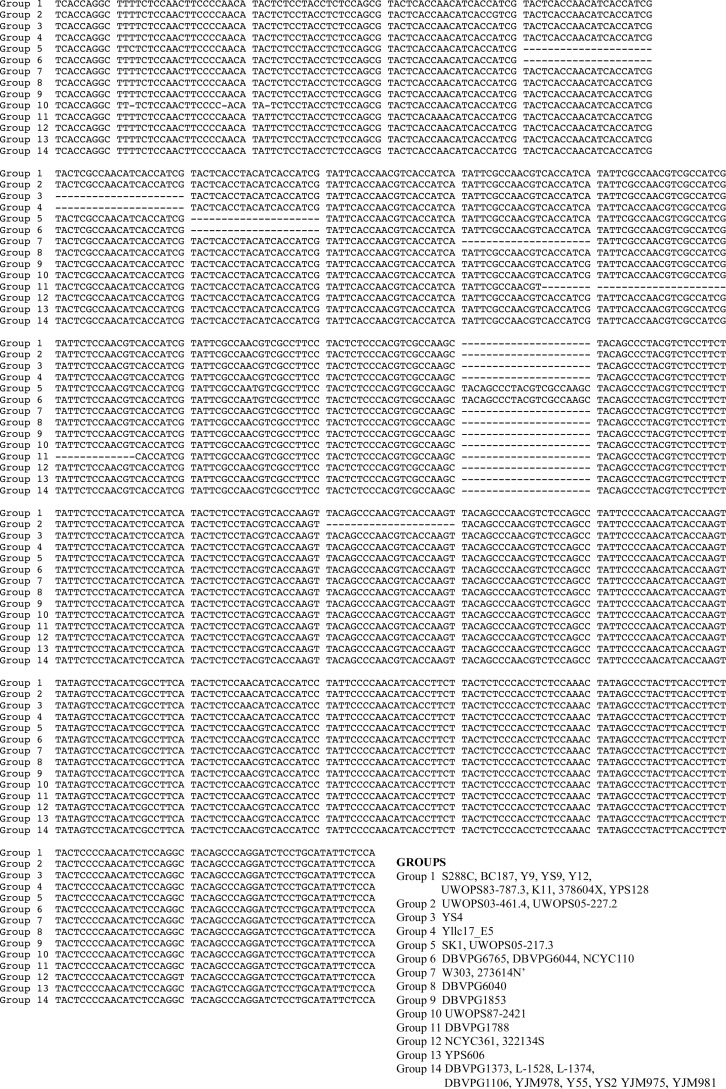
**Sequence alignment of the CTD region from 36 yeast strains from the Saccharomyces Genome Resequencing Project.** Strains are grouped by identity and numbered according to vertical position on the tree in Fig. 1. Alignments were made using MUSCLE and hand-corrected to account for misaligned repeats.

Many groups have investigated the evolution of the unusual repetitive sequence that comprises the CTD of RNAPII across organisms (20–22). We wanted to look more closely for evidence of recent expansion or contractions within this coding region and took advantage of strains available from the Saccharomyces Genome Resequencing Project (23). Because of overlapping reads implemented in Next generation sequencing, repetitive domains are often difficult to accurately characterize. Therefore, we resequenced the CTD region of 36 of these strains using Sanger sequencing. Careful multiple sequence alignment revealed that the 36 strains could be arranged into 14 different sequence groups based on sequence similarity (Fig. 1). Half of these groups were defined by the addition or subtraction of a 21-base pair repeat unit at different locations within the sequence, shown in Fig. 1 as gaps in the sequence. These gaps are evidence of a rearrangement with the CTD repeat coding sequence. There were 11 instances of single nucleotide polymorphisms across all 36 strains (shown in *orange* in Fig. 1). The full alignments can be found in Fig. 2.

##### Yeast Correct Mutations within the CTD Primarily through Contraction

**FIGURE 3.**
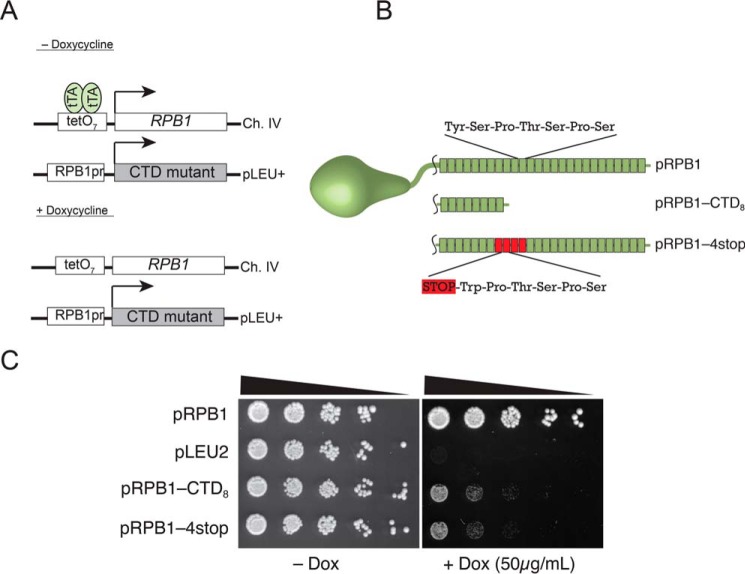
**A genetic system for measuring changes in CTD repeat length.** *A*, Tet-Off system for examining mutations within the CTD. In the absence of doxycycline, the Tet transactivator (*tTA*) binds to tetO_7_ sites upstream of the genomic copy of *RPB1* allowing transcription. In the presence of doxycycline, the genomic copy is effectively off, and cells rely on a plasmid-based copy of *RPB1* under the control of its endogenous promoter. *B*, schematic of mutant CTD constructs. Each block indicates the presence of one 21-bp repeat encoding the seven-amino acid CTD consensus sequence. pRPB1-CTD_8_ encodes a sequence with eight CTD repeats, whereas pRPB1–4stop encodes 26 repeats but is interrupted by four stop codons (in *red*). pRPB1–4stop encodes a protein with only seven functional CTD repeats. *C*, spotting assay demonstrating the effectiveness of our Tet-Off system. Both pRPB1-CTD_8_ and pRPB1–4stop produce a protein product that results in poor yeast viability in the presence of doxycycline. Spotting assays are representative examples of at least three independent trials.

**FIGURE 4.**
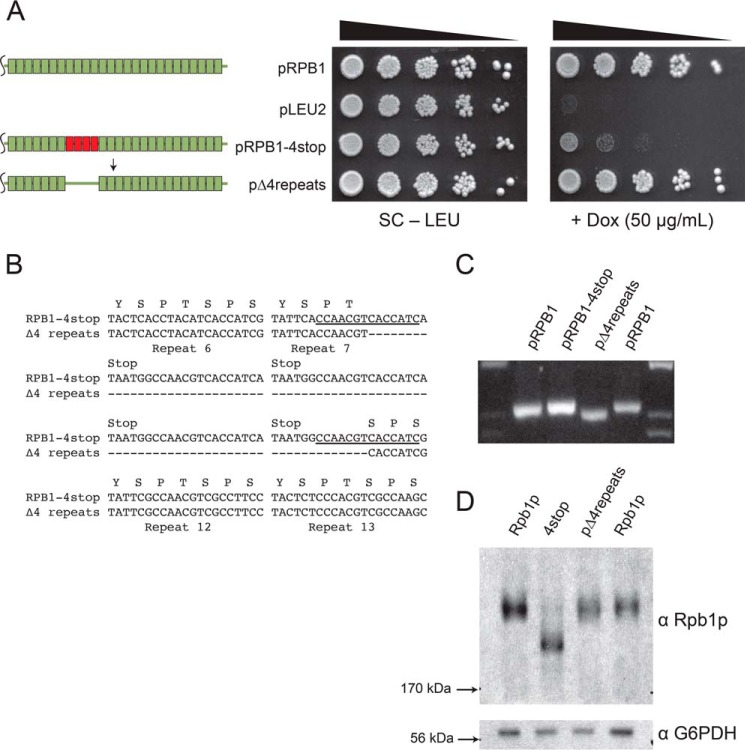
**The CTD coding region undergoes spontaneous contraction.** *A*, schematic depicting an *RPB1* mutant that spontaneously arises in cells with pRPB1–4stop. Plasmids isolated from fast growing colonies of GRY3019 transformed with pRPB1–4stop were retransformed into new isolates of GRY3019 to demonstrate that genetic changes to the plasmid were responsible for the phenotype. Retransformation of the Δ4 repeat suppressor into new cells resulted in improved growth on media containing DOX. Spotting assays are representative examples of at least three independent trials. *B*, sequence alignment of pRPB1–4stop and pΔ4repeats reveals that the contraction occurred between two regions of microhomology flanking the stop codons (*underlined*). *C*, PCR analysis of the same plasmids shown in *A* shows that DNA encoding pΔ4repeats is indeed smaller as observed on agarose gel. *D*, Western blot of extracts grown from the cells in *A* in the presence of DOX. The contraction event seen in the Δ4 mutation results in a protein product that is larger than RPB1–4stop. Key to visualizing small molecular weight differences in CTD mutants is the nonstandard acrylamide:bisacrylamide ratio (see “Experimental Procedures”). The data shown are a representative blot of three independent trials.

Sequencing of strains allowed us to uncover past instances of CTD rearrangement, but this does not tell us anything about the relative frequency at which these arrangements occur. To directly observe rearrangements occurring within the CTD coding sequence, we synthesized a variant of the CTD (pRPB1–4stop) in which repeats 8–11 each contained a stop codon Tyr^1^ → stop (Fig. 3*B*) and a noncoding Ser^2^ → Trp mutation. Previous studies had determined that 8 or more CTD repeats were required for efficient growth, and we predicted that this mutant RPB1–4stop gene would not be capable of supporting growth. To test this, we devised a Tet-Off expression system based off the work of Strathern and co-workers (9). Briefly, we introduced CTD variants on CEN/ARS-containing plasmids into yeast under the control of the native *RPB1* promoter. When appropriate, doxycycline was introduced to repress transcription of the genomic copy of *RPB1*, which was under the control of a tetracycline-responsive promoter (Fig. 3*A*). Under these conditions, we could force the cell to rely on the Rpb1p variant for transcription and monitor these effects by growth on SC−Leu plates containing DOX. As expected, the protein produced from our RPB1–4stop mutant harbors only 7 CTD repeats, and cells show extremely compromised growth comparable to a RPB1-CTD_8_, a CTD variant that contains only 8 repeats (Fig. 3*C*). The cells were grown to large colonies to allow for the accumulation of spontaneous mutations. When plated on medium containing doxycycline, after 3–4 days we observed the presence of colonies that were able to bypass the slow growth conferred by our reporter constructs. We expected these fast growing suppressors to result from four types of events: 1) contractions where the introduced stop codons were removed from the suppressor plasmids, 2) expansions where the region encoding the first 8 repeats was expanded to yield a protein product that had more than 8 repeats, 3) homologous recombination events where the mutant plasmid copy of *RPB1* underwent a rearrangement with the doxycycline-regulated copy of *RPB1* in the genome, and 4) mutations elsewhere in the genome. We performed a fluctuation analysis to measure the mutation frequency of this reporter and determined the relative proportion of different mutagenic events based on PCR analysis of numerous fast growing suppressors. To avoid potential bias for jackpot mutations, the cells plated on each fluctuation plate were derived from individual yeast colonies. Furthermore, we analyzed no more than four colonies from any one individual plate. The data are summarized in Table 3. Nearly half of the events we observed were the result of contractions that remove the stop codons (Fig. 4, *A* and *B*). Interestingly, for all contractions, the product yielded a protein sequence with the expected YSPTSPS sequence at all repeats. To confirm that the improved growth was due to the change in the plasmid, several suppressor plasmids were retransformed into GRY3019 and monitored for improved growth (Fig. 4*A*), CTD coding region length (Fig. 4*C*), and Rpb1p protein size by Western blot (Fig. 4*D*).

**TABLE 3:** **Analysis of CTD rearrangements** The rates of suppressor formation were determined by measuring the frequencies of fast growing colonies on SC−Leu plates containing doxycycline. Rates and 95% confidence intervals (in parentheses) were calculated using the MSS-maximum likelihood estimator method (16, 17).

Reporter	Rate × 10^−6^/cell/generation	Total	Expansion	Contraction	Recombination	Other	Expansion	Contraction
							%	%
pRPB1–4stop	26 (19–42)	115	1	59	ND*^a^*	55	<1	51
pRPB1-CTD_8_	4.5 (2.5–6.9)	169	65	0	15	89	38	0

*^a^* Homologous recombination events cannot be distinguished from extragenic suppressors by colony PCR so they are included into the “other” category.

##### Truncated CTD Variants Undergo Spontaneous Expansion

**FIGURE 5.**
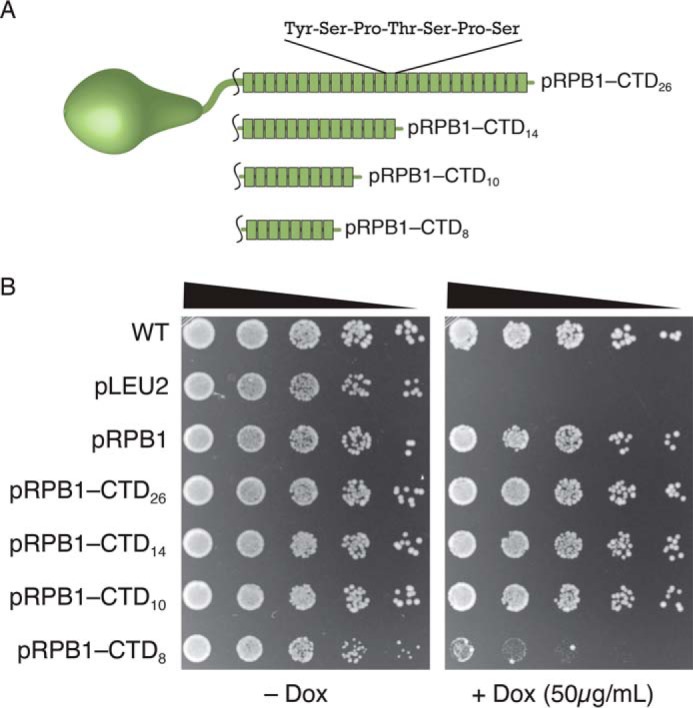
**Yeast viability is dependent on CTD length.** *A*, schematic depicting *RPB1* mutants with variable length CTDs. Each seven-amino acid (21 base pairs) repeat is shown as a single *green box. B*, strains expressing CTD mutants with as few as ten repeats show near wild-type growth; however, a mutant with only eight repeats is severely compromised as reported by others using the classic *URA3* shuffling system. Spotting assays are representative examples of at least three independent trials.

**FIGURE 6.**
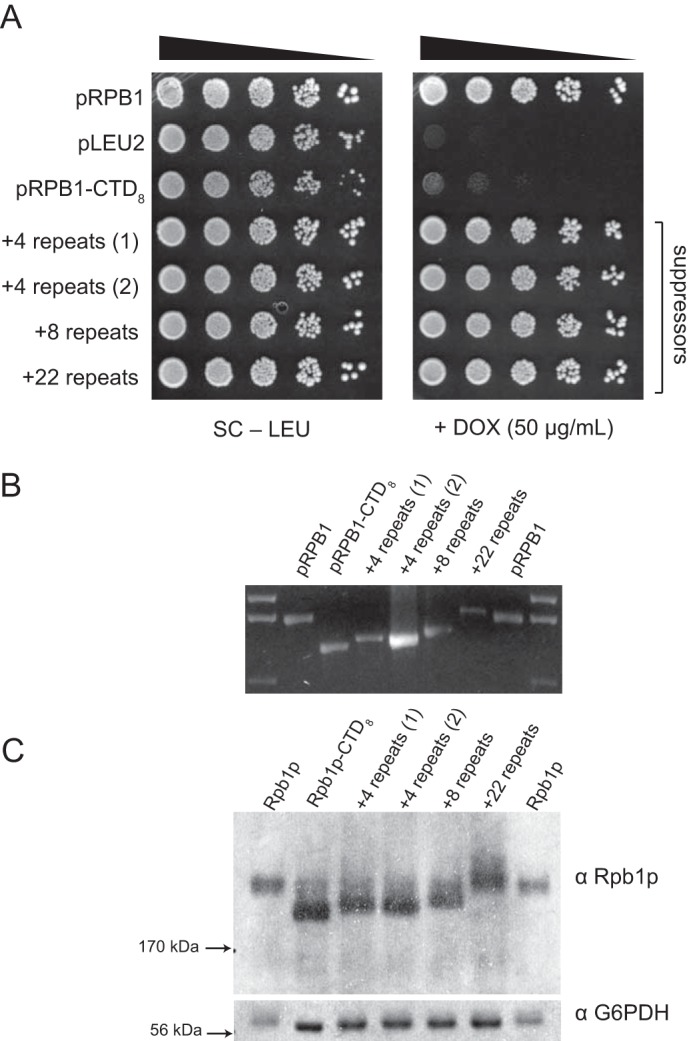
**Spotting analysis of several suppressors isolated from strains originally transformed with pRPB1-CTD_8_.** *A*, reintroduction of the plasmids from these suppressors resulted in drastically improved growth on medium containing doxycycline, confirming that genetic changes to pRPB1-CTD_8_ were responsible for the phenotype. Spotting assays are representative examples of at least three independent trials. *B*, PCR analysis of the plasmids from *A* shows that DNA encoding suppressors of pRPB1-CTD_8_ contain more CTD repeats. This was confirmed by Sanger sequencing (data not shown). *C*, Western analysis demonstrates that expression of *RPB1* from these suppressor plasmids results in protein products that are higher molecular weight than the original CTD_8_ product. Key to visualizing small molecular weight differences in CTD mutants is the nonstandard acrylamide:bisacrylamide ratio (see “Experimental Procedures”). The data shown are a representative blot of three independent trials.

We expected our pRPB1–4stop reporter construct to reveal both expansion and contractions within the CTD coding region; however, expansions were difficult to detect with this system because of the high frequency of contractions. We therefore set out to design a second reporter construct to measure expansion by simply removing repeats from the C-terminal end of the CTD. First we determined the CTD length required to support growth in our Tet-Off system. Under these conditions, we see that CTD variants with as few as 10 repeats grow normally on +DOX plates, whereas cells with 8 repeats show impaired growth (Fig. 5, *A* and *B*). We therefore measured the appearance of suppressor colonies using this pRPB1-CTD_8_ plasmid. For pRPB1-CTD_8_, we observed the appearance of fast growing suppressor colonies after 3 or 4 days of growth at 30 °C. We performed a fluctuation assay as described above to characterize the events that allowed cells to bypass our CTD truncation mutants (Fig. 6). We used colony PCR to characterize suppressor events as expansions, homologous recombination, or extragenic mutations, and determined the frequency of each event (Table 3). Using this construct, we observed that expansions occurred in approximately one-third of all colonies and were more common than homologous recombination with the genomic copy. Subsequent sequencing of expansion and recombination events revealed that the CTD undergoes expansion from 8 to up to 30 repeats, longer than the canonical wild-type sequence (Fig. 6, *B* and *C*).

##### G_4_-DNA in the CTD Coding Region

**FIGURE 7.**
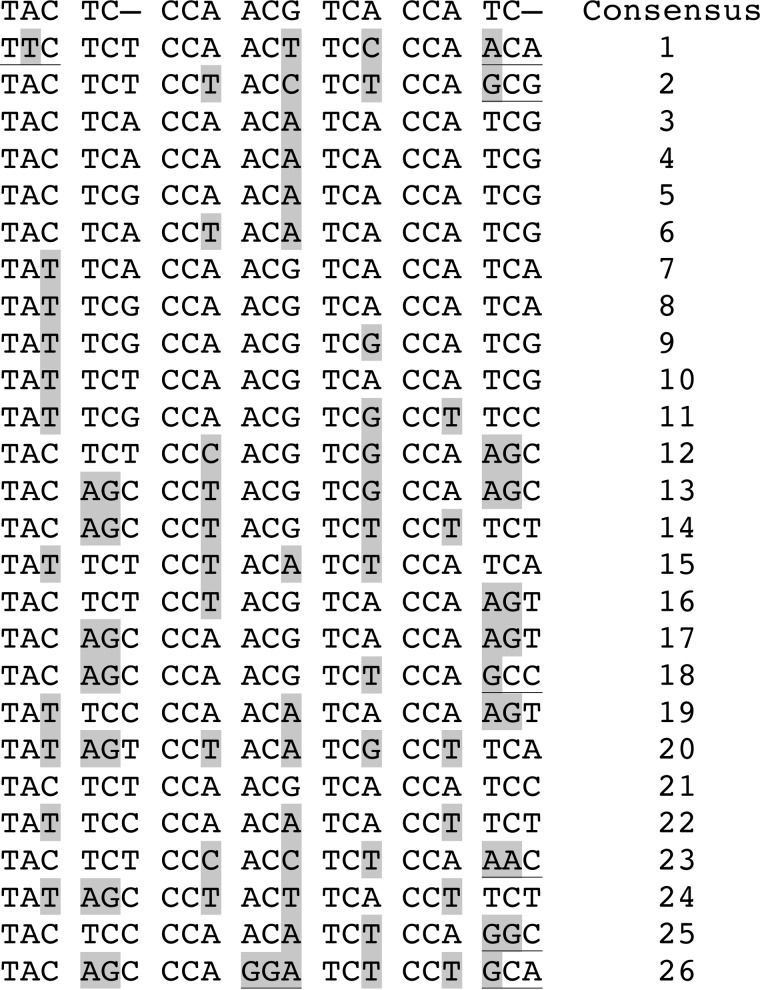
**Alignment of sequences for the individual CTD repeats.** Repeats are numbered according to their location in the DNA sequence. Bases that vary from the consensus (at *top*) are shaded in *gray*. Polymorphisms that result in an amino acid change are *underlined*.

**FIGURE 8.**
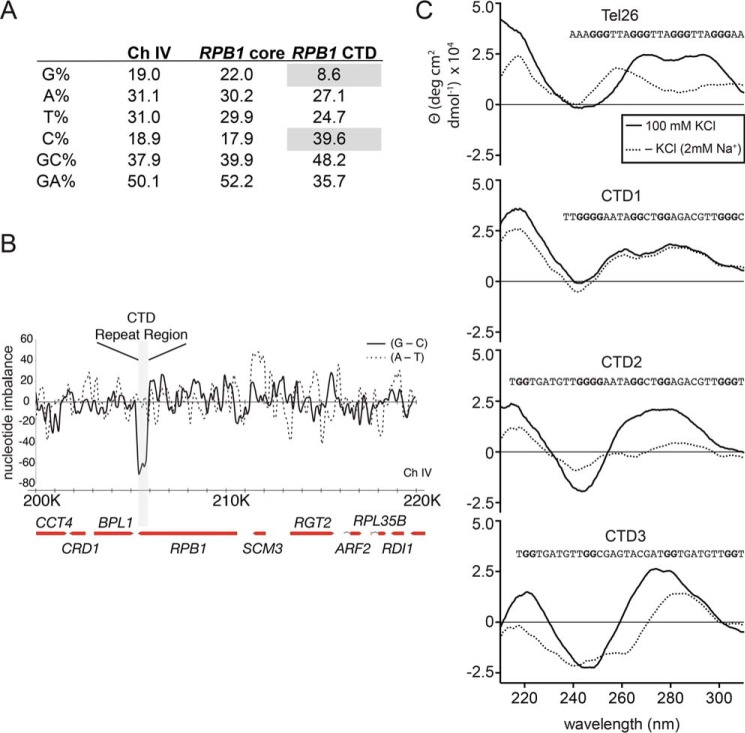
**GC imbalance marks the CTD coding region and contributes to secondary structure formation.** *A*, nucleotide statistics for the core of *RPB1* and the CTD region. The CTD coding strand has reduced guanine bases and a high concentration of cytosine (*gray*). *B*, plot of nucleotide differences for a 20-kilobase region of yeast chromosome IV including *RPB1*. The data were calculated from 150-base pair windows with 50 base pairs of overlap between adjacent windows and plotted in Excel. The CTD region has a very negative value of G-C. A-T for this same region is less variable. *C*, circular dichroism for G-rich DNA from the noncoding strand of the CTD identified as potential G4-DNA forming sequences using QGRS mapper and a known G4-DNA forming region of human telomeres (Tel26). The data were collected in the presence (*solid line*) and absence (*dotted line*) of 100 mm KCl. In the presence of potassium ions, all three oligonucleotides derived from the CTD coding sequence exhibit a minimum near 240 nm and a broad positive peak between 260 and 300 nm.

Expansion and contraction of trinucleotide repeat DNA is often mediated by slipped strands resulting in regions with DNA secondary structure (*e.g.* hairpins), which in turn involves the recruitment of various proteins associated with DNA repair processes. In yeast, the CTD coding consists of an imperfect 21-bp repeat where (for strain S288C) only two repeats share the same sequence (Fig. 7). Therefore, it is not intuitive how secondary structure might form to promote expansion and contraction processes. Capra *et al.* (24) recently reported G_4_-DNA near the 3′ end of the *RPB1* coding sequence. G_4_-DNA is known to promote DNA rearrangements in many organisms, including in the promoter of MYC in humans (8, 25). Although the rest of the coding region was not predicted to form additional G-quadruplex structures, we uncovered an unusual sequence feature within the CTD coding region, namely an increased frequency of cytosine bases on the coding strand (Fig. 8*A*). Conversely, the noncoding strand would be rich in guanine nucleotides, a feature known to promote secondary structure formation (26). Interestingly, this discrepancy in G:C ratio is restricted to the CTD coding region (Fig. 8*B*).

To test whether these sequences may be forming secondary structure, we used the QGRS Mapper (27) to identify candidate regions from the CTD coding region. We synthesized several single-stranded oligonucleotides corresponding to regions of the CTD coding sequence that have four consecutive tracks of at least two guanines and loop lengths of fewer than 10 bases. Indeed, by circular dichroism, these oligonucleotides formed stable structures (Fig. 8*C*) with a broad maxima between 265 and 300 nm and a minimum near 240 nm in the presence of potassium ions. In our hands, this is consistent with what has been reported for complex mixed parallel/antiparallel G_4_-DNA structures (28) such as that formed by human telomeres (Fig. 8*C*). G4-DNA structures generally require the presence of a cation (K^+^ > Na^+^). As such, we performed equivalent experiments in the absence of K^+^ (but still 2 mm Na^+^ from the buffer). Under these circumstances all CTD-derived DNAs still exhibited some structure, but there was little to no similarity among any of the DNA sequences under these conditions.

##### The DNA Helicase Pif1 Suppresses CTD Rearrangement

The Zakian lab (29) recently showed that the G_4_-DNA containing region within the CTD coding region also overlaps with genome association by the G-quadruplex unwinding helicase Pif1p. To test the role of Pif1p in mediating CTD expansion, we measured expansion and contractions in a *pif1-m2* mutant. This mutant disrupts Pif1p activity in the nucleus, and thus the mutant is predicted to have more difficulty resolving G_4_-DNA structures (11). Indeed, the percentages of suppressors using our pRPB1–4stop and pRPB1-CTD_8_ constructs were both increased in *pif1-m2* cells (Table 4). In particular, mutating *PIF1* increased expansion in pRPB1-CTD_8_ from 38% to 56% of all suppressor events.

**TABLE 4:** **Influence of Pif1p and Rad52p on CTD variability** Characterization of fast growing suppressors based on DNA sequence for two *RPB1* reporter constructs

	Expansions	Recombination	Other	Total	Expansions
					%
**pRPB1-CTD_8_**					
WT	65	15	89	169	38
*pif1-m2*	44	1	33	78	56
*rad52*Δ	6	0	115	115	5

**pRPB1-4stop**					
WT	59	1	55	115	51
*pif1-m2*	47	0	35	82	57
*rad52*Δ	51	0	1	52	98

##### Rad52 Is Required for Expansion of CTD Repeats

Much like expansions in microsatellite DNA, we hypothesized that CTD repeat length variation resulted from DNA damage repair pathways, primarily homologous recombination (HR). Accordingly, we measured expansions and contractions in recombination-deficient *rad52*Δ cells (Table 4). Expansions were essentially absent when *RAD52* was deleted. Surprisingly, in our contraction assay, we found that nearly all of the recovered pRPB1–4stop suppressors exhibited contraction, although the total number of suppressors identified was decreased in *rad52*Δ cells. We interpret these data to mean that HR is the preferred cellular pathway leading to suppressor mutants. However, HR only leads to length polymorphism when it results in an expansion. Conversely, contractions in our system must result from a recombination-independent mechanism that competes with HR.

## Discussion

The CTD of RNAPII has been the subject of scientific fascination for many years. Not only does it exhibit a highly conserved and highly repetitive protein sequence, but it also is the substrate for numerous post-translational modifications that dictate its function. The CTD has received much attention from an evolutionary perspective because the number and composition of seven-amino acid repeats differs so greatly between organisms (20, 30–32). Of particular interest to us was the fact that model organisms, such as budding yeast, have CTDs that are considerably longer than necessary for laboratory growth. Furthermore, historical observations by our lab and many others suggested that the CTD region, although essential, was also very prone to mutation. From these two observations, we reasoned that the nonessential CTD repeats may serve a function as templates for DNA repair. Here we explore that possibility by identifying some of the factors that contribute to CTD instability and variability.

We resequenced 36 yeast strains to properly characterize the underlying variability within the CTD coding region. Our initial evaluation of the sequence alignment suggested a high propensity of single-nucleotide polymorphism across this CTD coding region. However, careful re-examination of the DNA encoding each repeat following Sanger sequencing revealed numerous contraction events and evidence of the addition of at least one novel repeat. Repetitive minisatellites are prone to expand and contract (33–35). However, what differentiates the CTD of RNAPII is that this variable region encodes a conserved, essential region within an essential protein. Furthermore, the DNA encoding these repeats is in fact relatively dissimilar even though it encodes the same peptide sequence (Fig. 7). These factors make its instability worthy of additional study.

Our analysis of the Saccharomyces Genome Resequencing Project sequencing data supports a model where the CTD coding region spontaneously contracts or expands, resulting in a CTD length that could potentially alter fitness. Growth experiments from our lab and others clearly show that cells expressing very short CTDs (8–10 repeats) are less fit than cells with what is considered a full-length CTD (3, 36). Nonetheless, this initial finding suggested that yeast have the ability to explore different CTD repeat lengths. Previous studies from West and Corden (36) and Young and co-workers (3, 4) clearly demonstrate that cells expressing CTDs with at least 12 repeats show near wild-type growth under a range of growth conditions. Therefore it is interesting that the CTD copy number is variable in yeast but has fixed at a number of repeats much greater than what is required for viability.

We tested our model by developing two different genetic reporters: one that predominantly measured CTD contraction (pRPB1–4stop) and another that measures expansion (pRPB1-CTD_8_). Both took advantage of a very powerful doxycycline-regulated *RPB1* system developed by the Strathern lab and utilized the inability of Rpb1p with short CTDs to support normal yeast growth. This system turns out to be extremely important for the quantitation of suppressor events. Previous studies with CTD mutants have been conducted using the *URA3* shuffle system (4, 36y, 37), which requires a round of 5-fluoroorotic acid selection to cure cells of a wild-type *RPB1* plasmid. During this time, the cells are prone to fast growing suppressor mutations. By using a small molecule controlled system, we can switch off wild-type expression in already growing cells, reducing the likelihood of spurious mutation events.

Using our reporters, we were able to identify three broad classes of fast growing suppressors: expansions/contractions (changes in length of the CTD as measured by PCR), homologous recombination events with the genomic copy of *RPB1*, or mutations elsewhere in the genome that supported growth but have yet to be characterized. We were able to show by reintroduction of isolated suppressor plasmids that rearrangements of the DNA that led to constructs expressing more than eight wild-type CTD repeats were sufficient to confer improved growth. This additional large class of mutations elsewhere in the genome can presumably bypass our selection system through a number of mechanisms including altering *RPB1* expression (or copy number), altering sensitivity to DOX, or facilitating transcription elongation. In fact, Young and co-workers (38) used a similar system to initially identify members of the Mediator complex more than 20 years ago. The increased flexibility and sensitivity of our DOX system may facilitate future identification of additional regulators of transcription.

The rate of suppressor accumulation for both reporters was more than 1 × 10^−6^/cell/cell division, with the rate in our contraction assay being 10-fold higher than our expansion assay. Even though the DNA repeat of the CTD is a degenerate 21-bp repeat, this preference for contraction over expansion is consistent with other reports of instability within repetitive DNA sequences (39). This also explains the low frequency of expansions seen with the pRPB1–4stop construct, as it would likely be necessary to screen several hundred colonies to identify relatively rare expansion events among the more common contractions.

Mainly from studies of microsatellite DNA, several models have been developed to explain DNA expansion and contraction (34, 40). DNA secondary structure formation is important for many types of microsatellite instability including GAA and CAG repeats. In these models, stable secondary structures block processes, such as replication, leading to a break in the DNA that must be repaired. It is during this repair that sequence is lost (contraction) or reamplified (leading to expansion). Most of these studies have been performed on repeat sequences of fewer than 6 bp. The sequence encoding the CTD is unusual in that it is comprised of a 21-bp repeat and is nonidentical in the native sequence. Nonetheless, we noted that there is a feature of this sequence that supports secondary structure formation, namely an imbalance in the number of cytosine bases relative to guanine bases (225:44 C:G) on the coding strand. The high density of guanine bases in the CTD noncoding strand is able to form a stable secondary structure that resembles G_4_-DNA structures by circular dichroism (Fig. 8). This phenomenon was first described in minisatellite regions of the human genome (41), and G-rich strands from microsatellites such as the CGG repeat from *FMR1* (fragile X mental retardation 1) and the CAGGG repeat from Ms6-hm were subsequently shown to form secondary structure (26, 42). Likewise, these regions exhibited extremely high instability with mutation rates greater than 1% (26, 43). Interestingly, many but not all imbalanced minisatellites studied to date have been in noncoding regions. Furthermore, their base repetitive unit is generally less than 10 base pairs in length. How the CTD coding sequence may have evolved to have such strong imbalance remains a mystery. However, it is intriguing that each repeat encodes two prolines, ensuring a regular repeating CCN sequence every 6 or 9 bp, invariably across all 26 repeats and heavily skewing the GC balance. Loop length between polyguanine tracks has been shown to be crucial for the structure and stability of G-quadruplexes, with shorter and uniform loop lengths being preferred. We measured the structure of several oligonucleotide sequences derived from the CTD-coding region using circular dichroism. Nearly all formed structures despite nonuniform loop length and at least one loop of more than 7 bp. The exact structure of these oligonucleotides is still unknown. However, because of the large number of G tracks within the CTD, there is a possibility of secondary structure formations at dozens of sites within the CTD region.

This leads to a model for instability wherein the strong bias in single strand nucleotide composition within a region, rather than repetitive DNA, could promote instability. As expected, mutation of *PIF1*, a G_4_-DNA specific helicase, indeed increased the percentage of expansions in our system. It also drastically decreased the percentage of homologous recombination events with the genomic copy of *RPB1*. This can be explained based on recent work in which Pif1p is required for efficient recombination-coupled DNA repair (44). This also suggests HR as a probable pathway mediating CTD coding region instability. To begin to test this, we deleted *RAD52*, a central player in several branches of DDR. As expected, expansion events were nearly eliminated in *rad52*Δ, confirming a role for recombination in this process. Deletion of *RAD52* decreased the overall number of suppressors isolated but increased the percentage of contraction events. We interpret these results to mean that homologous recombination is necessary for expansion but competes for the mutagenic process of contraction. This also means contraction must occur through a recombination-independent pathway. It is not uncommon for multiple DNA repair processes to be recruited to the same site, because this has been shown for minisatellite instability in yeast (45) and double-stranded break repair in *Drosophila* (46). Sequence analysis of suppressor mutants isolated from the RPB1–4stop construct show that the deletions tend to unite sites of microhomology on both sides of the stop codon (Fig. 4*B*). There are many types of microhomology-mediated end joining (47), and thus future studies may be able to decipher which repair processes and additional helicases contribute to observed differences in expansion and contraction within the CTD coding region.

In conclusion, we believe the results described here highlight the instability inherent within the RNAPII CTD coding sequence. Furthermore, we provide a rationale for how both DNA secondary structure and DNA repair processes may contribute to this variability. This phenomenon is particularly significant because the CTD of *RPB1* is essential for viability in yeast. We suspect few other essential genes show such nucleotide level variability across individual members within the same species. Future studies will determine whether other tandem repeat-containing proteins are regulated by similar mechanisms.

## Author Contributions

S. M. F. conceived and coordinated the study and wrote the paper. S. A. M. designed, performed, and analyzed the experiments shown in Figs. 5 and 6 and Tables 3 and 4 and contributed to the preparation of the figures and the writing and editing of the original manuscript. A. E. performed and analyzed the experiments shown in Figs. 4 and 6 and Tables 3 and 4. M. B. performed and analyzed the experiments associated with Figs. 4 and 6. B. I. R. designed, performed, and analyzed the experiments shown in Figs. 1 and 2 and Table 4. All authors contributed to the preparation and editing of the manuscript.

## Footnotes

4 The abbreviations used are:

RNAPII: RNA polymerase II
CTD: C-terminal domain
G_4_-DNA: G-quadruplex DNA
DOX: doxycycline
HR: homologous recombination.
